# Traumatic Injuries Associated With Standing Motorized Scooters

**DOI:** 10.1001/jamanetworkopen.2020.1925

**Published:** 2020-03-31

**Authors:** Frank Bauer, Jeffrey D. Riley, Karen Lewandowski, Kaveh Najafi, Helen Markowski, John Kepros

**Affiliations:** 1 HonorHealth Scottsdale Osborn Medical Center, Scottsdale, Arizona; 2 Independent Researcher, Scottsdale, Arizona

## Abstract

This cross-sectional study assesses the incidence and severity of traumatic injuries associated with motorized scooters, as well as the associated use of protective devices and intoxicants.

## Introduction

Standing motorized scooters are increasingly used for personal transportation,^1^ yet such devices confer considerable risk for trauma, particularly to the head and extremities.^2,3,4,5^ As potential dangers are not widely known and training is often minimal, riders may often use these devices in an unsafe manner, eg, without protective gear and/or while intoxicated.^2^ This single-center study assessed the incidence and severity of traumatic injuries associated with motorized scooters and the associated use of protective devices and/or intoxicants.

## Methods

Following HonorHealth Institutional Review Board approval, a retrospective, single-institution, cross-sectional study via review of deidentified medical records (deemed exempt from informed consent) was conducted to determine the number of patients with scooter-associated injury at the American College of Surgeons Level 1 HonorHealth Scottsdale Osborn Emergency Department (ED) from October 1, 2018, to October 1, 2019. Associated trauma activations were triaged according to severity of trauma (Figure) (red = high, yellow = moderate, and green = low or nonactivation). Data collected included demographic characteristics, medical history, injury type and location, Injury Severity Score, use of protective devices, alcohol and toxicology screening results, medical treatment, ED disposition, imaging and treatments required, and intensive care unit (ICU) care. Descriptive statistics included frequencies and percentages. This study followed the Strengthening the Reporting of Observational Studies in Epidemiology (STROBE) reporting guideline.

**Figure.**
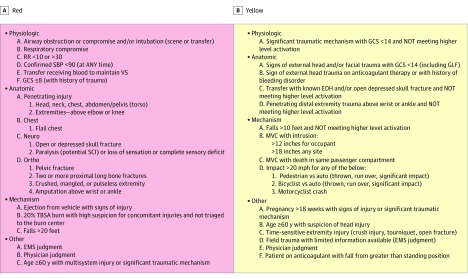
Triage Level Criteria for Trauma Activations Patients are triaged according to physiologic, anatomic, mechanism of injury, and/or other criteria. Individuals meeting any one of the criteria are placed in that triage level. EDH indicates epidural hematoma; EMS, emergency medical services; GCS, Glasgow Coma Scale; GLF, ground-level fall; MVC, motor vehicle crash; RR, respiratory rate; SBP, systolic blood pressure; SCI, spinal cord injury; TBSA, total body surface area; and VS, vital signs.

## Results

We identified 61 patients (mean [SD] age, 33.6 [13.0] years; 26 women [43%]) with scooter-related injuries over the study duration (total number of trauma activations = 2070). No incidents involved the use of protective devices at time of injury. The mean (SD) Injury Severity Score was 6.3 (6.0); 2 deaths from traumatic brain injury were recorded. Injury characteristics and ED disposition are presented in the Table. The most common injuries were to the head, face, and/or neck (n = 51 [84%]); 22 of these patients also had extremity injuries. No visceral organ injuries or episodes of hemorrhagic shock were noted. Twenty patients received ICU care, all but 2 of whom had severe head, neck, and/or facial injuries and/or neurological injuries (15 were transferred directly from the ED, and 5 were transferred during their hospital stay). Although a majority of injuries (n = 59 [97%]) were associated with single-rider or single-vehicle incidents, 2 of the severe incidents involved automobiles. Thirty-seven patients tested positive for an intoxicating substance; specifically, 21 tested positive for alcohol, 7 for illicit drug use, and 9 for both.

**Table.** Injuries, Injury Severity, and Emergency Department Disposition Following Motorized Scooter Accidents

Characteristic	No. (%)
Injury Severity Score (n = 61)	
Mild (1-8)	45 (74)
Moderate (9-14)	8 (13)
Severe (16-24)	7 (11)
Critical (25-27)	1 (2)
Triage level (n = 61)	
Green	8 (13)
Yellow	43 (70)
Red	3 (5)
Orthopedic consultation	7 (11)
Injury type and location (nonexclusive)	
Fracture (n = 43)	
Head, face, and/or neck	22 (51)
Arm	6 (14)
Torso	10 (23)
Leg	5 (12)
Wound (n = 70)	
Head, face, and/or neck	35 (50)
Arm	15 (21)
Torso	6 (9)
Leg	14 (20)
Traumatic brain injury (n = 17)	
Concussion	9
Intracerebral hemorrhage	8
Loss of consciousness (n = 61)	
No	33 (54)
Yes	28 (46)
Disposition from emergency department (n = 61)	
Admitted to floor	23 (38)
Home without services	18 (30)
Operating room	3 (5)
Intensive care unit	15 (25)
Left against medical advice	1 (2)

## Discussion

Standardized motorized scooters are an increasingly popular mode of transportation,^6^ but emerging evidence suggests that their use may be associated with an increased risk of injury.^3,4,5,6^ The present study found that scooter-related injuries are highly associated with lack of protective devices and that many injuries occur in the context of alcohol and/or illicit drug use. Although predominantly mild in severity, a minority of injuries were severe and life threatening.

This study has some limitations. Of note, this was a single-center, retrospective study with a short time frame, including only patients seen by a trauma surgeon in the ED, and therefore may undercount injuries, particularly if minor in severity. This limitation, along with the fact that our institution does not have an intermediate care unit, may explain the relatively high number of patients admitted to the ICU. In line with previous studies,^2,3^ we found that the use of protective devices appears to be exceedingly rare; indeed, no individuals were found to be using a protective device at time of injury. Furthermore, the use of alcohol and/or illicit drugs was a significant risk factor for injury, consistent with previous findings.^2^ Taken together, these findings suggest a gap in public awareness of the potential dangers of standing motorized scooters.

We believe further study is warranted to help formulate public policy and that public awareness of the risk associated with these vehicles needs to be expanded. Furthermore, trauma activation criteria may need to be updated to reflect an increasingly common mechanism of injury.
